# Meta-analysis of hemodynamic optimization: relationship to methodological quality

**DOI:** 10.1186/cc3902

**Published:** 2005-11-15

**Authors:** Martijn Poeze, Jan Willem M Greve, Graham Ramsay

**Affiliations:** 1 Department of Surgery, University Hospital Maastricht, P Debyelaan 25, 6202 AZ Maastricht, The Netherlands

## Abstract

**Introduction:**

To review systematically the effect of interventions aimed at hemodynamic optimization and to relate this to the quality of individual published trials.

**Methods:**

A systematic, computerized bibliographic search of published studies and citation reviews of relevant studies was performed. All randomized clinical trials in which adult patients were included in a trial deliberately aiming at an optimized or maximized hemodynamic condition of the patients (with oxygen delivery, cardiac index, oxygen consumption, mixed venous oxygen saturation and/or stroke volume as end-points) were selected. A total of 30 studies were selected for independent review. Two reviewers extracted data on population, intervention, outcome and methodological quality. Agreement between reviewers was high: differences were eventually resolved by third-party decision. The methodological quality of the studies was moderate (mean 9.0, SD 1.7), and the outcomes of the randomized clinical trials were not related to their quality.

**Results:**

Efforts to achieve an optimized hemodynamic condition resulted in a decreased mortality rate (relative risk ratio (RR) 0.75 (95% confidence interval (CI) 0.62 to 0.90) in all studies combined. This was due to a significantly decreased mortality in peri-operative intervention studies (RR 0.66 (95% CI 0.54 to 0.81). Overall, patients with sepsis and overt organ failure do not benefit from this method (RR 0.92 (95% CI 0.75 to 1.11)).

**Conclusion:**

This systematic review showed that interventions aimed at hemodynamic optimization reduced mortality. In particular, trials including peri-operative interventions aimed at the hemodynamic optimization of high-risk surgical patients reduce mortality. Overall, this effect was not related to the trial quality.

## Introduction

It has been shown that, in critically ill patients, impaired cardiovascular function has a role in the development of organ failure. Our understanding of the underlying mechanism responsible for this dysfunction has changed over the past 10 years. Previously, correction of disturbed hemodynamics to normal values in the peri-operative phase was considered standard care in the treatment of surgical patients. However, clinical signs of hypovolemia are non-specific and non-sensitive [1]. Moreover, because the mean values of commonly used parameters, such as central venous pressure and pulmonary artery occlusion pressure, are similar between survivors and non-survivors, the value of correcting these parameters to normal values is questionable [2]. The same is true for critically ill patients treated for sepsis at an intensive care unit [1].

A report by Shoemaker and colleagues [3] changed the prevailing views on the hemodynamic treatment of the critically ill patient. In this report the authors observed that 'normal' values are 'abnormal' in post-operative, trauma and critically ill patients. In comparison with non-surviving patients, surviving trauma patients had above-normal oxygen delivery and oxygen consumption values. These 'supra-normal' values may reflect an ability of these patients to respond adequately to the 'stress' of the trauma.

There have been a considerable number of randomized, controlled, clinical studies investigating the role of improving patients' hemodynamic condition by increasing oxygen delivery to the tissues to supranormal levels or by other goals. Heyland and colleagues published a review in 1996 evaluating studies that included patients for whom supranormal oxygen delivery was the goal of treatment [4]. This review, including a total of 1,291 patients, found no difference in outcome but identified a relation between outcome and trial quality [4]. In two recent meta-analyses, Kern and Shoemaker [5] and Boyd and Hayes [6] found a significant reduction in mortality, but they did not report data on quality analysis.

We therefore decided to perform a systematic review of the effects of interventions aimed at hemodynamic optimization and to examine their relation to the quality of the individual published trials. We hypothesized that a reduced trial quality would be related to a greater reported survival difference.

## Materials and methods

### Study identification

Three methods were used to retrieve information for this review [7,8]. First, MEDLINE and EMBASE databases for the years 1980 to 2005 were searched, with the following mesh headings: 'oxygen consumption' or 'hemodynamics' or 'dobutamine' or 'fluid therapy', exploding with 'randomized controlled trials' (publication type) and 'intensive care', 'critical care' or 'intensive care unit' or 'surgery' or 'peri-operative care'. The second method used was to search personal files and communications to find additional citations and to search *Current Contents *for recently published studies. Third, the reference lists of the articles found with the above-mentioned methods were searched for additional articles.

### Study selection

The articles found using this search method were classified into original articles, reviews and others (such as letters). Studies were selected if they involved a randomized controlled trial with fluid and/or additional vasoactive therapy to optimize or maximize the hemodynamic condition of the patients (end-points: oxygen delivery, cardiac index, oxygen consumption, mixed venous oxygen saturation and/or stroke volume). Moreover, the studies included had to have been performed either among an adult intensive care unit population or an adult surgical population. Studies with zero mortality in both treatment arms were not excluded from the meta-analysis.

### Methodological quality assessment

A methodological scoring system (Table 1) was used to give a relative assessment of the quality of the primarily selected studies [9]. The scoring system was based on the system proposed and validated by Chalmers [9] and previously used by Heyland and colleagues [4]. The scores for the individual studies were compared between two independent observers, and in the event of disagreement a third (non-involved) person decided on the score assigned to the study. Because not all studies aimed at the reduction of mortality as a primary end-point, a scoring distinction was made between studies aiming primarily at reducing mortality (two points) and those having a reduced mortality as a secondary end-point (one point). The presence of crossover is defined as a patient achieving the hemodynamic goals of the opposite group from that to which he or she had been allocated (that is, a patient in the control group achieving the oxygen delivery goal defined for the treatment group, without additional treatment).

### Statistical analysis

Data are shown as percentages or absolute numbers ± SD. A statistical meta-analysis was performed with Review Manager 4.2. The primary outcome was the overall mortality rate reported at 28 to 30 days. The relative risk ratios for the individual studies and the overall relative risk ratios with 95% confidence intervals (CIs) were calculated by means of the method developed by Mantel and Haenszel. To assess the heterogeneity between studies, we used the method developed by DerSimonian and Laird [10]. If no significant heterogeneity was found, a fixed-effects model was used to calculate pooled relative risk and 95% CIs.

Several subset analyses were performed. One subset analysis compared the results for 'peri-operative' and 'sepsis' patients included in the various studies. The two patient groups (peri-operative patients and patients with sepsis and organ failure) were separated by using the inclusion criteria from the original studies, based on pathophysiological differences [11]. This subset therefore differentiates between the effects of optimization techniques in peri-operative patients and in patients with organ failure or sepsis and organ failure. The hypothesis tested in this subset analysis was that hemodynamic optimization to values above normal improves the outcome in peri-operative patients (including post-traumatic patients), but has no effect in patients with sepsis and organ failure.

A second subset analysis included the studies using the original 'supranormal' hemodynamic optimization criteria proposed by Shoemaker and colleagues (that is, cardiac index > 4.5 l min^-1 ^m^-2^, oxygen delivery > 600 ml min^-1 ^m^-2 ^or oxygen consumption (VO_2_) > 170 ml min^-1 ^m^-2^) [3,12-28]. The other studies used a variety of therapeutic goals, including mixed venous oxygen saturation (SvO_2_) [22,29-31], left-ventricle stroke work index [32], stroke volume [33,34], or cardiac index values lower than 4.5 l min^-1 ^m^-2 ^[35-40]. For the purpose of this subset analysis, the study by Gattinoni and colleagues [22] was divided into two datasets. One included the patients for whom cardiac index was the goal of treatment. This dataset was included in the subset of studies using the original criteria proposed by Shoemaker and colleagues [3]. The patients for whom SvO_2 _was the goal of treatment were included in the other study subset.

In addition, subset analyses were conducted to investigate the effects of the methodological quality criteria. One subset analysis compared studies having a quality score above 10, indicating adequate trial quality, with those having a quality score below 10. This cutoff value for the methodological quality was determined from the peak incidence of quality scores. Finally, the individual quality items of using the presence of mortality as an end-point, blinding and crossover were tested separately in a subset analysis.

## Results

### Study inclusion and allocation

After initial screening and a subsequent more detailed evaluation of retrieved randomized trial reports, 32 candidate trials were identified. A total of 30 studies were included in the analysis. Two studies were omitted from the analysis after careful review of the methodology: the study by Garrison and colleagues [41] was a case-control study, and the study by Blow and colleagues [42] used no randomization. Of the 30 remaining trials, 21 involved surgery or trauma patients who were hemodynamically optimized peri-operatively, and 9 involved patients with sepsis and/or organ failure.

### Study results

The total number of patients included in the studies was 5,733. The median number of patients who were randomized was 75 (range 30 to 1,994; Tables 2 and 3). The mean score on the methodological quality assessment in the included studies was 9.1 (95% CI 7 to 12.7), which is 57% of the maximum score of 16. The duration of follow-up, up to 28 or 30 days, was specified in all trials. Other characteristics of the trials are shown in Tables 2 and 3.

**Figure 1:**
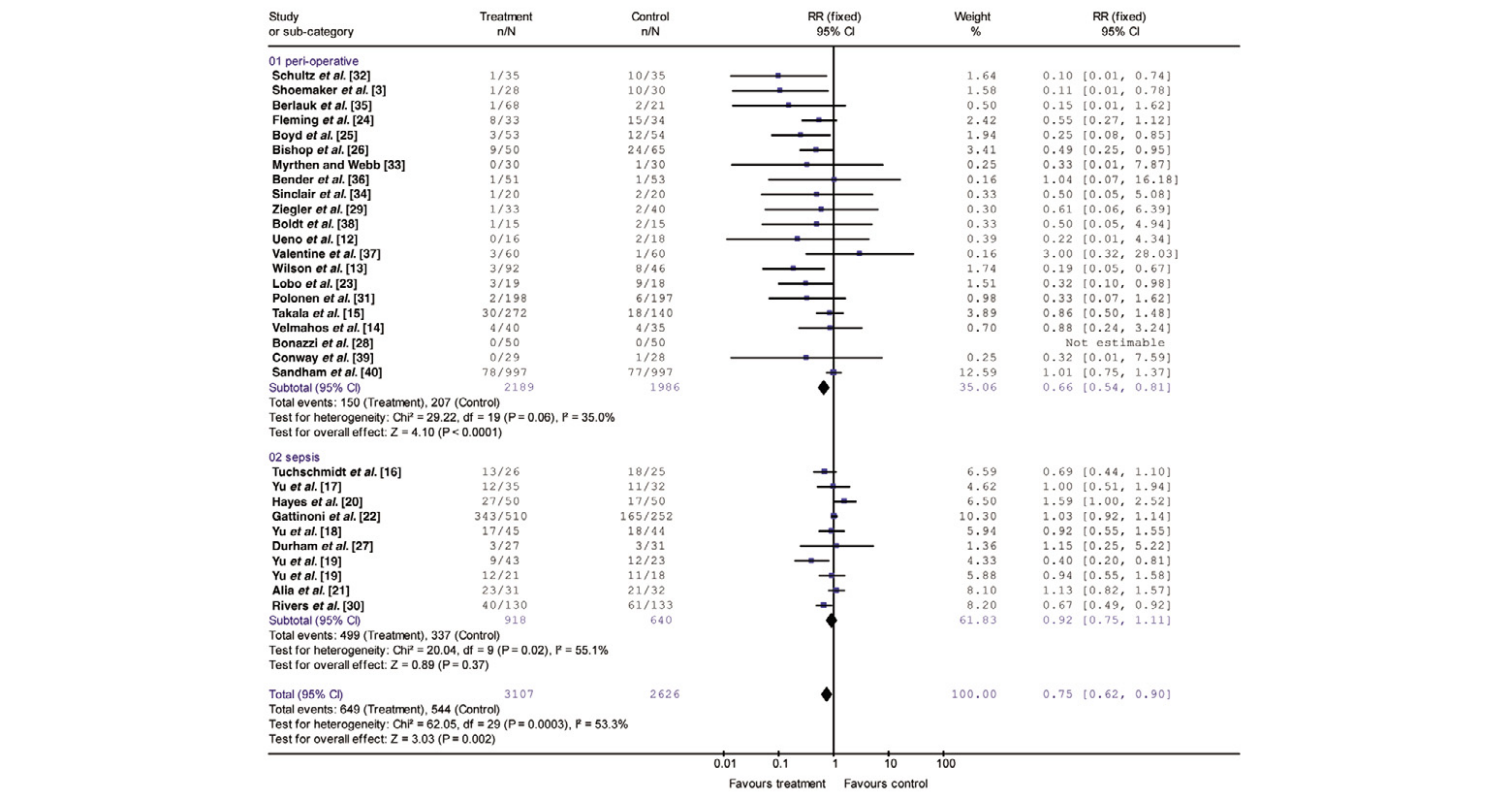
Relative risk determined in individual trials in studies (including subset analysis with patients treated peri-operatively and patients with sepsis and/or organ failure) shown as boxes scaled according to weighting, using the inverse variance method. Error bars indicate 95% confidence intervals (95% CI). A fixed-effects model (peri-operative studies) was used when heterogeneity analysis was not significant, and a random-effects model (sepsis and total included studies) was used when heterogeneity analysis was significant. The pooled relative risk estimates are shown as diamonds that span the 95% CI. *n*, number of deceased patients in the treatment or control arm; *N*, total number of patients in treatment or control arm; RR, relative risk ratio.

The odds ratio for all studies combined was 0.61 (95% CI 0.46 to 0.81) with a relative risk of 0.75 (95% CI 0.62 to 0.90; Figure 1). However, the absolute risk reduction was only 0.4% (95% CI -1.7 to 2.6%). Moreover, of the 30 studies included, only 8 showed a significantly greater survival in the optimized patients, whereas one study showed a significantly greater mortality in the optimized patient group, and the other studies did not show a significant difference in survival. For quality control, we correlated the score of the quality assessment with the odds ratio for the individual studies. This correlation was not significant (*r *= 0.33; *p *= 0.07).

### Subset analysis

#### Peri-operative and trauma studies versus studies using septic/organ failure patients

There were 4,174 patients enrolled in the studies that used strategies to optimize the hemodynamic condition peri-operatively and during trauma (Table 2). The overall odds ratio for mortality with hemodynamic optimization in this group was 0.43 (95% CI 0.28 to 0.66) with a relative risk ratio of 0.66 (95% CI 0.54 to 0.81; Figure 1). Of the 21 studies, 6 showed a significantly reduced mortality in the treatment group. When using an optimization protocol, 31 patients (95% CI 20 to 63) had to be treated to save one life. The number of patients that must be included in a single study to be able to find this difference is 500, assuming a mortality rate of 15% in the control group.

The overall odds ratio for the 1,558 enrolled patients with septic shock/organ failure was 0.85 (95% CI 0.58 to 1.25) with a relative risk ratio of 0.92 (95% CI 0.75 to 1.11; Figure 1 and Table 3). Of the 10 included studies, 3 found either a tendency towards increased mortality or a significantly increased mortality in the treated patients. Two studies found an improved survival.

The mean quality score for the peri-operative studies did not differ from the mean score for the studies of septic/organ failure patients (9.0 ± 1.9 versus 9.0 ± 1.3; *p *= 0.9). Neither the peri-operative studies nor the studies including patients with sepsis had a significant correlation between the score and the odds ratio (*r *= 0.28, *p *= 0.3, and *r *= 0.28, *p *= 0.4, respectively).

#### Supranormal oxygen delivery as a goal of treatment

Our analysis for all studies combined, but only including those patients optimized by using the criteria proposed by Shoemaker (total number of included patients; *n *= 2,181), yielded an odds ratio of 0.60 (95% CI 0.42 to 0.88), with a relative risk ratio of 0.75 (95% CI 0.60 to 0.95). This significant effect was not found in the patient group for whom supranormal oxygen delivery was not used as the end-point (relative risk ratio 0.81 (95% CI 0.62 to 1.07); Table 4).

The subgroup analysis of the peri-operative studies that included individual studies using the original criteria proposed by Shoemaker (with 1,142 patients) found a relative risk ratio of 0.41 (0.29 to 0.59; Table 4). In these studies, 10 patients (95% CI 7 to 16) needed to be treated to save one life. The quality control score of this subgroup was 9.1 (SD 2.5). Studies using treatment goals other than supranormal oxygen delivery in peri-operative patients found no effect on mortality; the relative risk ratio was 0.84 (0.64 to 1.10).

In the studies including patients with sepsis and organ failure, neither the use of supranormal oxygen delivery nor other specified treatment goals yielded a reduction in mortality; relative risk ratios were 1.00 (95% CI 0.90 to 1.11) and 0.93 (95% CI 0.83 to 1.05), respectively (Table 4).

#### Quality assessment score

Studies with a high quality assessment (a score of 10 or more) tended to report a higher relative risk ratio, although the difference was not significant (mean 0.84; 95% CI 0.66 to 1.07) than studies with a lower quality assessment score (mean 0.60; 95% CI 0.48 to 0.75; Table 4). In the subset of studies including peri-operative and trauma patients, the overall outcome was not related to the trial quality. The studies with a quality score of 10 or more found a relative risk ratio of 0.60 (95% CI 0.38 to 0.95), compared with a relative risk ratio of 0.27 (95% CI 0.13 to 0.55) in the studies with a quality score of less than 10. Other cutoff points were also tested but produced similar results (data not shown).

#### Mortality end-point

Relative risk ratios were calculated for 29 of the 30 included studies. In the combined studies that had mortality as the primary end-point, the effect on mortality tended to be lower than that in the remaining studies, although the difference was not significant (Table 4).

#### Blinding

Only three studies (10%) randomized patients with adequate blinding. The effect on mortality was not significantly different in studies with inadequate blinding from that found in the studies without blinding (Table 4).

#### Crossover

In the studies in which crossover between the treatment arms was adequately controlled for, no significant effect on mortality was found in comparison with the studies with significant crossover (Table 4).

## Discussion

This meta-analysis, for which we conducted a systematic search, selection and quality assessment of the literature, suggests that optimization techniques can improve survival when used in peri-operative and trauma patients without sepsis or multiple organ failure. Overall, patients with sepsis and overt organ failure do not benefit from this method.

The use of hemodynamic optimization as a therapy to improve outcome is controversial. The regimen was originally designed to optimize the hemodynamic status in high-risk surgical patients. The initial studies found an improved outcome, although doubt remained about the methodological quality of these studies. A large number of studies, using different patient populations and optimization techniques, were subsequently conducted. A considerable number of these studies found no improved outcome [14,16,22]. Moreover, one study found an increased mortality rate in the optimized patient group [20]. The meta-analysis by Heyland and colleagues [4], reporting the first seven studies published at that time, found no overall benefit from maximizing oxygen delivery with the aim of improving outcome. This meta-analysis also criticized the quality of the individual studies. A subsequent meta-analysis by Kern and Shoemaker found a significantly lower mortality in patient groups optimized at an early stage (namely surgical patients optimized peri-operatively), but no formal quality analysis was presented [5]. Our meta-analysis represents the most up-to-date evaluation of the issue of hemodynamic optimization in which a quality assessment was performed and related to the outcome of the studies. It suggests that hemodynamic optimization strategies are beneficial in all patient subgroups but that the overall effect is explained by the significant improvement in mortality in those studies including peri-operative and trauma patients.

There are several critical issues to be addressed before valid conclusions can be drawn from the present meta-analysis. The overall trial quality has been called into question previously [4] and we found in our meta-analysis that studies with a high trial quality score (using the cutoff point of 10 out of 16) did not report an improved mortality rate. Fortunately, the trial quality seemed to influence the outcome in the studies including peri-operative patients less than the outcome in the subset of patients with established sepsis and multiple organ failure. In addition, the largest effect on mortality was found in the studies including peri-operative patients.

Another critical point may be the cutoff point that we chose to divide the individual studies between those with a high quality score and those with a low score. However, other cutoff points that we tested produced similar data (data not shown). Moreover, we also tested the effect of individual trial quality features on the outcome of the studies included. Thus, although the overall trial quality is moderate it may be concluded that the impact of this on the outcome of the meta-analysis is not significant.

One may question our subset analysis, which divided the studies into a subset with studies involving peri-operative and trauma patients and one with studies using septic/organ failure patients. It has been suggested, both in reviews [43,44] and in a previous meta-analysis [6], that the outcome of studies with late interventions should be separated from those of studies with early interventions. A similar distinction was made in the meta-analysis by Heyland and colleagues [4]. In addition, in the meta-analysis by Kern and Shoemaker [5] risk differences (-0.23 ± 0.07) in the subgroup using goals to supranormal values in patients before organ failure were found comparable to the data reported in our study (risk difference -0.12; 95% CI -0.20 to -0.03). Recent publications have indeed reported a pathophysiological basis for this distinction. In an early stage of the disease process of the systemic inflammatory response syndrome, it is possible to prevent or overcome peripheral defects in oxygen delivery, on the basis of decreased flow, hypoxia or hypovolemia. In contrast, persistent defects in oxygen delivery to the tissues during decreased flow or hypovolemia may alter vascular and cellular metabolism. These defects in cellular oxygenation become irreversible as a result of mitochondrial damage, and when they occur in the endothelium they lead to vascular hyporeactivity or 'vasoplegia', resulting in impaired perfusion and organ failure. Moreover, organ function is less likely to recover at this stage because of the relative insensitivity of patients with multiple organ failure to the optimization techniques [11,45,46]. The study by Rivers and colleagues [30], in which early optimization of the hemodynamics led to a reduced mortality even in patients with early septic shock, underlines this point.

The studies in our meta-analysis included different patient groups with varying co-morbidities and expected mortality rates. The differentiation between the patients included in early and late intervention studies partly compensates for this effect. Some studies had a lower statistical power than expected, because of the low mortality rate in control patients. In the study by Takala and colleagues [15], no survival benefit was found in the overall study group, which had low baseline mortality, but a survival benefit was detected in a subgroup with higher baseline mortality (namely emergency surgery).

## Conclusion

There is sufficient evidence that aiming for optimized oxygen transport values in patients with high-risk surgery or trauma is beneficial and that the trial quality, although overall only moderate, is not important in these patients. The promising results obtained by Rivers and colleagues [30] and the aggressive early optimization of sepsis deserves further investigation and confirmation. However, patients with established organ failure due to sepsis do not benefit from attempts to optimize oxygen transport values.

## Key messages

• 	Peri-operative interventions aimed at the hemodynamic optimization of high-risk surgical patients reduce mortality.

• 	The use of hemodynamic optimization as a therapy to improve outcome during sepsis with established organ failure does not reduce mortality.

• 	Although there is no clear relationship between overall trial quality and outcome of all trials, individual quality assessment items influenced study outcome.

## Abbreviations

CI = confidence interval; RR = relative risk ratio; SvO_2 _= mixed venous oxygen saturation; VO_2 _= oxygen consumption.

## Competing interests

The authors declare that they have no competing interests.

## Authors' contributions

MP carried out the study gathering, scored the individual trials, participated in its design and coordination and wrote the manuscript. JG participated in its design and coordination and helped to draft the manuscript. GR carried out the study gathering, scored the individual trials, participated in its design and coordination and helped to draft the manuscript. All authors read and approved the final manuscript.

## Figures and Tables

**Table 1:** Quality control criteria for methodology of the studies

	Score		
	
Criterion	0	1	2
Method			
Randomization	Not randomized		Randomized
Blinding	Not blinded		Double-blind
Analysis	Other		Intention-to-treat
End-point mortality	No mortality as end point	Secondary end-point	Primary end-point
Population			
Patient selection	Selected patients or unclear	Consecutive eligible patients	
Comparability at baseline	No or unclear	Yes	
Extent of follow-up	Incomplete	Complete	
Intervention			
Treatment protocol	Unclear	Reproducible	
Co-interventions	Not described	Described, but not equal or unclear	Well described and equal
Crossover	Not described	>10%	<10%

**Table 2:** Attributes of included trials with peri-operative patients

Study	Population	Intervention	Blinding	Allocation concealment	Co-interventions	Crossover	Mortality end-point	Score	Goals of treatment
Schultz *et al*. 1985 [32]	Hip fractured patients	Fluids and inotropes peri-operatively	No	Adequate	Not described	Unclear	Yes	8	LVSW/PCWP optimized according to normogram
Shoemaker *et al*. 1988 [3]	High-risk surgical patients	Fluids and inotropes begun pre-operatively	No	Inadequate	Not described	Unclear	Yes	5	CI > 4.5, DO_2 _> 600, VO_2 _> 170
Berlauk *et al*. 1991 [35]	Peripheral vascular surgical patients	Fluids, afterload reduction and inotropes	No	Adequate	Not described	Unclear	Yes	9	CI > 2.8, 8 < PCWP < 15, SVR 1,100
Fleming *et al*. 1992 [24]	Trauma patients	Fluids, blood and dobutamine	No	Inadequate	Not described	>10%	Yes	7	CI > 4.5, DO_2 _> 670, VO_2 _> 166
Boyd *et al*. 1993 [25]	High-risk surgical patients	Fluids and dopexamine	No	Adequate	Described, but not equal	Unclear	Yes	10	DO_2 _> 600
Bishop *et al*. 1995 [26]	Cardiac surgical patients	Fluids and dobutamine	No	Adequate	Not described	>10%	Yes	10	CI > 4.5, DO_2 _> 670, VO_2 _> 166, PCWP 18
Mythen and Webb 1995 [33]	Cardiac surgical patients	Fluids	No	Adequate	Not described	Unclear	Yes	8	SV optimized
Bender *et al*. 1997 [36]	Elective vascular surgical patients	Fluids, blood, vasodilators, nitroprusside and dopamine	No	Adequate	Not described	Unclear	Yes	8	8 PCWP 14, CI 2.8, SVR 1,100
Ziegler *et al*. 1997 [29]	Elective vascular surgical patients	Fluids, blood, inotropes and vasodilators	No	Adequate	Not described	Unclear	Yes	9	SvO_2 _> 65, PCWP > 12, Hb > 10
Sinclair *et al*. 1997 [34]	Hip fractured patients	Fluids	No	Adequate	Not described	>10%	Yes	8	SV optimized to 0.35 < FTc < 0.40
Valentine *et al*. 1998 [37]	Elective aortic surgical patients	Fluids, nitroprusside, nitroglycerine and dopamine	No	Adequate	Not described	Unclear	Yes	10	CI > 2.8, 8 PCWP 15, SVR 1,100
Ueno *et al*. 1998 [12]	Elective hepatic surgical patients	Fluids and dobutamine	No	Adequate	Not described	Unclear	No	7	CI > 4.5, DO_2 _> 600, VO_2 _> 170
Boldt *et al*. 1998 [38]	Pancreatic surgical patients	Dopexamine	Yes	Adequate	Not described	Unclear	No	8	MAP 70, CI > 2.5, 12 < PCWP < 14
Wilson *et al*. 1999 [13]	High-risk surgical patients	Dopexamine or noradrenaline	Yes	Adequate	Described, but not equal	Unclear	Yes	12	DO_2 _> 600
Lobo et al 2000 [23]	High-risk surgical patients	Fluids and dobutamine	No	Adequate	Described, but not equal	>10%	Yes	11	DO_2 _> 600
Velhamos *et al*. 2000 [14]	Trauma surgical patients	Fluids, blood, inotropes and vasopressors	No	Adequate	Not described	>10%	Yes	11	CI > 4.5, DO_2 _> 600, VO_2 _> 170, SpO_2_/FiO_2 _> 200
Polonen *et al*. 2000 [31]	Cardiac surgical patients	Fluids, blood and inotropes	No	Adequate	Not described	>10%	Yes, but secondary	7	SvO_2 _> 70, lactate levels < 2.0
Takala *et al*. 2000 [15]	High-risk surgical patients	Fluids, blood and dopexamine	Yes	Adequate	Not described	>10%	Yes	13	DO_2 _> 600
Bonazzi *et al*. 2002 [28]	Elective vascular surgical patients	Fluids, inotropes, vasodilators	No	Adequate	Adequate	Unclear	No	10	CI > 3.0, 10 < PCWP < 18, SVR < 1,450, DO_2 _> 600
Conway *et al*. 2002 [39]	Elective gastro-intestinal surgical patients	Fluids	No	Inadequate	Not described	Unclear	Yes	8	CO optimized
Sandham *et al*. 2003 [40]	High-risk surgical patients	Fluids, blood, inotropes, vasodilators, vasopressors	No	Adequate	Not described	<10%	Yes	11	550 < DO_2 _< 600, 3.5 < CI < 4.5

CI, cardiac index (l min^-1 ^m^-2^); DO_2_, oxygen delivery (ml min^-1 ^m^-2^); FTc, corrected flow time; Hb, haemoglobin; LVSW, left ventricular stroke work; MAP, mean arterial pressure (mmHg); PCWP, pulmonary capillary wedge pressure; SpO_2_/FiO_2_, ratio of oxygen saturation as measured by pulse-oximetry and inspiration oxygen fraction; SV, stroke volume (ml); SvO_2_, mixed venous oxygen saturation (%); SVR, systemic vascular resistance (dyn s^-1 ^cm^-5^); VO_2_, oxygen consumption (ml min^-1 ^m^-2^).

**Table 3:** Attributes of included trials involving patients with sepsis and organ failure

Study	Population	Intervention	Blinding	Allocation concealment	Co-interventions	Crossover	Mortality end-point	Score	Goals of treatment
Tuchschmidt *et al*. 1992 [16]	Septic shock patients	Fluids, inotropes	No	Inadequate	Not described	>10%	Yes	9	CI > 6, SAP > 90
Yu *et al*. 1993 [17]	Sepsis, septic shock, ARDS patients	Fluids, blood, inotropes	No	Inadequate	Not described	>10%	Yes	8	DO_2 _> 600
Hayes *et al*. 1994 [20]	Post-operative patients, sepsis, respiratory failure	Fluids, dobutamine	No	Adequate	Not described	Unclear	Yes	10	CI > 4.5, DO_2 _> 600, VO_2 _> 170
Gattinoni *et al*. 1995 [22]	High-risk postoperative patients, sepsis, respiratory failure	Fluids and inotropes	No	Adequate	Described, but not adequate	<10%	Yes	12	CI > 4.5 or SvO_2 _> 70%
Yu *et al*. 1995 [18]	Sepsis, septic shock, ARDS or hypovolemic shock patients	Fluids, inotropes and vasopressors	No	Inadequate	Not described	>10%	Yes	8	DO_2 _> 600
Yu *et al*. 1998 [19]	SIRS, sepsis, severe sepsis, septic shock, ARDS patients 50–75 years of age	Fluids, afterload reduction, inotropes, amrinone, vasopressors	No	Adequate	Not described	Unclear	Yes	8	DO_2 _> 600
Yu *et al*. 1998 [19]	SIRS, sepsis, severe sepsis, septic shock, ARDS patients >75 years of age	Fluids, afterload reduction, inotropes, amrinone, vasopressors	No	Adequate	Not described	Unclear	Yes	8	DO_2 _> 600
Durham *et al*. 1996 [27]	Critically ill patients	Fluids, inotropes and nitroprusside	No	Adequate	Not described	Unclear	Yes	9	DO_2 _> 600, VO_2 _> 150
Alia *et al*. 1999 [21]	Septic shock patients or severe sepsis patients	Dobutamine	No	Adequate	Not described	>10%	Yes	10	DO_2 _> 600
Rivers *et al*. 2001 [30]	Severe sepsis and septic shock	Fluids, blood, inotropes and vasopressors	No	Adequate	Not described	>10%	Yes	11	SvO_2 _> 70%

ARDS, acute respiratory distress syndrome; CI, cardiac index; DO_2_, oxygen delivery; SAP, systolic arterial pressure; SIRS, systemic inflammatory response syndrome; SvO_2_, mixed venous oxygen saturation; VO_2_, oxygen consumption.

**Table 4:** Subset analyses of pooled relative risk of death

Trial category	Odds ratio (95% CI)	Relative risk (95% CI)	*p *for heterogeneity
Peri-operative trials			
Cardiac index, DO_2 _or VO_2_	0.41 (0.29–0.59)	0.49 (0.36–0.65)	0.1
Other goals	0.83 (0.62–1.11)	0.84 (0.64–1.10)	0.3
Sepsis/organ failure trials			
Cardiac index, DO_2 _or VO_2_	1.00 (0.77–1.30)	1.00 (0.90–1.11)	0.09
Other goals	0.77 (0.38–1.57)	0.85 (0.55–1.31)	0.01
All trials			
Cardiac index, DO_2 _or VO_2_	0.60 (0.42–0.88)	0.75 (0.60–0.95)	0.0003
Other goals	0.83 (0.68–1.03)	0.90 (0.80–1.01)	0.09
Score			
≥10	0.84 (0.66–1.07)	0.74 (0.51–1.08)	0.0005
<10	0.45 (0.32–0.64)	0.60 (0.48–0.75)	0.6
End-point			
Mortality as primary end-point	0.63 (0.46–0.85)	0.76 (0.63–0.93)	<0.0001
Secondary or no mortality end-point	0.34 (0.13–0.93)	0.36 (0.14–0.94)	1.0
Blinding			
Yes	0.61 (0.36–1.04)	0.64 (0.40–1.03)	0.1
No	0.62 (0.46–0.84)	0.77 (0.63–0.93)	0.0008
Crossover			
Yes	0.43 (0.25–0.77)	0.52 (0.32–0.83)	0.003
No	0.85 (0.71–1.01)	0.86 (0.73–1.02)	0.05

Risk analyses comparing subset including the use of hemodynamic goals with supranormal values (cardiac index, DO_2_, or VO_2_) or with other goals both in all trials included, in peri-operative trials, and in studies including patients with sepsis and established organ failure. Risk analysis was also calculated in the subgroup of studies with a quality assessment score of 10 or more, comparing them with the studies with a quality assessment score of less than 10. Individual quality assessment items were also analysed for risk reduction, including the use of mortality as primary end-point in the studies, the use of adequate blinding, and the presence of crossover phenomena. A fixed-effects model for calculating the odds ratio and relative risk ratio was used when heterogeneity analysis (last column) was not significant. 95% CI, 95% confidence interval; DO_2_, oxygen delivery; VO_2_, oxygen consumption.
